# Active Edible Films Based on Arrowroot Starch with Microparticles of Blackberry Pulp Obtained by Freeze-Drying for Food Packaging

**DOI:** 10.3390/polym11091382

**Published:** 2019-08-23

**Authors:** Gislaine Ferreira Nogueira, Farayde Matta Fakhouri, José Ignacio Velasco, Rafael Augustus de Oliveira

**Affiliations:** 1 School of Agricultural Engineering, University of Campinas, Campinas SP 13083-875, Brazil; 2 Centre Català del Plàstic, Dpt. of Materials Science and Metallurgy, Universitat Politècnica de Catalunya, Carrer Colom 114, Terrassa E-08022, Spain; 3 Faculty of Engineering, Federal University of Grande Dourados, Dourados MS 79804-970, Brazil

**Keywords:** blackberry, arrowroot starch, gum arabic, freeze-drying, water solubility, water vapor permeability, anthocyanins, antioxidant capacity, powder, food packaging

## Abstract

This research work evaluated the influence of the type of incorporation and variation in the concentration of blackberry pulp (BL) and microencapsulated blackberry pulp (ML) powders by freeze-drying on the chemical and physical properties of arrowroot starch films. Blackberry powders were added to the film-forming suspension in different concentrations, 0%, 20%, 30% and 40% (mass/mass of dry starch) and through two different techniques, directly (D) and by sprinkling (S). Scanning electron microscopy (SEM) images revealed that the incorporation of blackberry powder has rendered the surface of the film rough and irregular. Films incorporated with BL and ML powders showed an increase in thickness and water solubility and a decrease in tensile strength in comparison with the film containing 0% powder. The incorporation of blackberry BL and ML powders into films transferred colour, anthocyanins and antioxidant capacity to the resulting films. Films added with blackberry powder by sprinkling were more soluble in water and presented higher antioxidant capacity than films incorporated directly, suggesting great potential as a vehicle for releasing bioactive compounds into food.

## 1. Introduction

The production of active edible films for active edible packaging is gaining interest from researchers and the industry due to their potential to control the quality and stability of many food products (dried fruits, meat and fish, among others) [1,2]. Current research on active packaging has been focusing on the encapsulation of natural bioactive compounds, antimicrobial and antioxidant agents, vitamins, aromas and dyes, within biodegradable packaging materials [1,2,3,4,5,6,7,8,9,10,11,12]. This approach can improve protection properties and also generate custom properties, such as antioxidants and antimicrobials, innovative flavours, aromas and colours [4,5,6].

Due to these aspects, countless research projects are being conducted in this direction. For instance, the incorporation of green tea extract into chitosan films [7]; red raspberry extract, rich in anthocyanins, into isolated soy protein films [8]; natural extract of beet and carrot into hydroxypropyl methylcellulose films [9]; oil resin, oregano, olive, pepper, garlic, onion and cranberry into chitosan films; and casein into carboxymethyl cellulose films [10], among many other studies.

Films containing blackberry pulp presented anti-inflammatory activity and increased cell viability [11]. The use of blackberry in films is promising and deserves further exploration, since this fruit is an excellent agricultural resource for combining nutritional properties and biological activities in the same food, which can bring benefits both to the food in which it will be packed, due to its antioxidants, and to the consumer in terms of nutrition. Blackberry is a rich source of antioxidant compounds, such as phenolic acids, tannins and anthocyanins [13,14].

Several studies have reported higher antioxidant capacity in blackberries based on their oxygen radical absorbance capacity in comparison with other fruits, such as strawberries, red raspberries and red wine grapes [15,16,17]. In addition, the microencapsulation of blackberry pulp as a possibility to maintain the stability of its antioxidant compounds when exposed to unfavourable conditions (e.g., high temperature, light, oxygen) is a viable alternative and a promising technology to preserve its functionality [18].

Among the techniques of microencapsulation, freeze-drying is the most used in the food industry; low temperatures are applied, which favour the preservation of bioactive materials that are sensitive to high temperatures [19,20]. Freeze-drying is a technique based on dehydration by sublimation of a frozen product. During this procedure, blackberry pulp is homogenized with the encapsulating agent and then co-lyophilized, obtaining in the end a material with a dry aspect [21], which can be easily reduced to powder with microparticles with diameters of μm-mm [22]. 

The incorporation of microparticles of blackberry into the film can protect antioxidant compounds, and allow their release, in a controlled manner, into the food during storage. In addition, the way of incorporating these microparticles influences their location within the film matrix and this may influence this release, as well as the physico-chemical properties (microstructure, mechanical and barrier properties, colour, anthocyanins content and antioxidant capacity) presented by the film [23].

Recently, Nogueira, Fakhouri and Oliveira [24] reported methods used for the preparation of starch films incorporated, directly and by sprinkling, with dried blackberry microparticles. In the direct incorporation method, the blackberry powder was added directly into the filmogenic solution and later deposited onto the support plate for drying; whereas, in the incorporation method by sprinkling, the filmogenic solution was previously deposited onto the support plate and, subsequently, the blackberry powder was sprinkled with a stainless steel sieve onto its surface. According to the authors, the resulting films were homogeneous and the incorporation of blackberry powder into the films did not make them sticky; instead, they could be easily manipulated. Most of the blackberry particles remained intact after their direct incorporation, as well as by sprinkling, into the film-forming suspension, since it was possible for the naked eye to visualize particles in the resulting dry film. Moreover, using the sieve to sprinkle the powder onto the film-forming solution allowed the blackberry particles to fall evenly across each surface area of the film. Nogueira, Fakhouri and Oliveira [23] confirmed this behaviour through scanning electron microscope (SEM) images, in which particles added by sprinkling into the filmogenic solution tend to remain on the surface, differing from particles added directly into the filmogenic solution, which tend to be more integrated in the resulting film matrix. The fact that the particles are located on the surface of the film allows greater contact with the food or aqueous media, resulting in a faster release of the particles and solubilization of the film. When the film is consumed along with the food, it should be able to release encapsulated compounds to the food system or during passage into the gastrointestinal tract after consumption [23,25]. For active edible films, high solubility in aqueous media is a desirable feature [25].

The incorporation of encapsulated blackberry pulp into the film can promote a controlled release of the antioxidant compounds onto the food surface during storage, where it will act to prevent oxidation and the formation of undesirable food flavours and textures [26,27,28]. In the work carried out by Talón et al. [27], sunflower oil oxidation was prevented when using films incorporated with encapsulated eugenol. Pork belly packaged with Job’s tears starch film containing 0.5% clove bud essential oil exhibited a lower degree of lipid oxidation determined by peroxide and thiobarbituric acid reactive substances than a non-packaged sample during storage [28]. 

Given the great potential as active packaging, further studies are still necessary for a better understanding of the influence of blackberry microparticles on properties of arrowroot starch films. This knowledge will be important in the future to more efficiently develop active films incorporated with blackberry microparticles and for their eventual application as active food packaging and partial substitutes for non-biodegradable plastic packaging used for specific food wrappers such as sushi, or to be consumed as fruit strips as a source of nutritional compounds. 

Thus, the aim of this research work was to develop edible and active arrowroot starch films incorporated, directly (D) and by sprinkling (S), with blackberry pulp (BL) and microencapsulated blackberry pulp (ML) powders by freeze-drying. The influence of the method of incorporation and the variation in the concentration of blackberry powders in properties of starch films were investigated. The blackberry powders were characterized with regard to drying process, moisture content, water activity, hygroscopicity, solubility, microstructure, colour, anthocyanins content and antioxidant properties. The films were characterized regarding microstructure, colour, anthocyanins content, antioxidant properties, thickness, water activity, moisture content, water solubility, water vapor permeability and mechanical properties.

## 2. Materials and Methods 

### 2.1. Materials

In this work, we used frozen blackberries (*Rubus fruticosus*), cv. Tupy, containing total solid content of 10.3 g/100 g of pulp (Agro Monte Verde Eirelli”, MG, Brazil), gum arabic InstantgumVR (colloids Naturels, São Paulo, Brazil) containing 14.00% ± 0.10% of moisture content, 1.38% ± 0.16% of proteins, 0.37% ± 0.02% of lipids, 3.79% ± 0.10% of ash and 80.46% ± 0.10% of carbohydrates [29], arrowroot starch containing 15.24% ± 0.19% of water, 0.40% ± 0.03% of protein, 0.12% ± 0.01% of fat, 0.33% ± 0.01% of ash, 83.91% ± 0.10% of carbohydrates [29] and amylose content of 35.20% ± 1.63% [30] and glycerol P.A. (Reagen, Quimibrás Indústrias Químicas S.A.- Rio de Janeiro, Brazil) as plasticizing agent. All other reagents used for the analysis presented analytical grade.

### 2.2. Production of Blackberry Pulp (BL) and Microencapsulated Blackberry Pulp (ML) Powders by Freeze-Drying

The production of blackberry pulp (BL) and microencapsulated blackberry pulp (ML) powders by freeze-drying followed the methodology described by Nogueira, Fakhouri and Oliveira [23]. A portion of frozen blackberry pulp with and without encapsulating agent (encapsulating agent consisting of arrowroot starch and arabic gum mixture (1:1 mass/mass) in ratio of 1:1.78 (mass/mass, blackberry pulp solids for encapsulating agent) was homogenized in a homogenizer mixer at room temperature for 5 min, and freeze-dried (Mod. 501, Edwards Pirani, Crawley, West Sussex, UK), with initial temperature of −40 °C, pressure of 0.1 mmHg and final temperature of 25 °C per 2 h, with total cycle time of 48 h. The resulting dried product was ground in a hammer mill (MR Manesco and Ranieri Ltd.a, model MR020, Piracicaba, Brazil) and sieved (28 mesh). The powders were stocked in hermetic pots, coated with aluminium foil to protect against photodegradation and, subsequently, stored in desiccators containing dried silica gel at 25 °C.

#### Characterization of Blackberry Powder

The blackberry powders were characterized with regard to drying process as the ratio between powder solid mass and the mass of total solids in the feed solution, in triplicate. The moisture content of powders was gravimetrically determined, in triplicate, by drying the samples in a vacuum oven at 60 °C until constant weight [29]. For the determination of water activity, AquaLab Lite apparatus (Decagon Devices Inc., Pullman, WA, USA) was used, by direct reading of the samples, in triplicate, at 25 °C. 

Hygroscopicity was determined following the methodology described by Cai and Corke [31], with modifications. Samples (about 1 g in Petri dishes) of each powder were placed, in triplicate in desiccators containing saturated sodium chloride (NaCl) solution (75.7% relative humidity at 25 °C). After one week, each sample was weighed and hygroscopicity was expressed as grams of water absorbed per 100 g of dry solids. 

For the determination of solubility, the method proposed by Eastman and Moore (1984), cited by Cano-Chauca, Stringheta, Ramos and Cal-Vidal [32] was followed. One gram of each sample was added to 100 mL of distilled water and maintained at high stirring speed in the magnetic stirrer for 5 min. Then, the solution was centrifuged at 3000 G for 5 min. An aliquot of 25 mL of the supernatant was transferred to pre-weighed Petri dishes and submitted to drying in the oven at 105 °C until constant weight. Solubility (%) was calculated by weight difference.

### 2.3. Incorporation of Blackberry Powders into Film-Forming Solution

#### 2.3.1. Preparation of Film-Forming Solution

Film-forming solution was prepared by dispersing arrowroot starch in distilled water (4%, mass/mass), as optimized by Nogueira, Fakhouri and Oliveira [33], and heated at 85 °C in a thermostatic bath, under constant agitation, for about 5 min. Then, glycerol was added to the starch solution at a concentration of 17% (mass/mass of total solids, as optimized by Nogueira, Fakhouri and Oliveira [33]) and homogenized. Blackberry powders were added to the film-forming solution in concentrations of 0%, 20%, 30% and 40% (mass/mass of total solids). This incorporation of blackberry powder into the film-forming solution was performed in two ways, directly (D) and by sprinkling (S), following the methodology proposed by Nogueira, Fakhouri and Oliveira [24]. 

#### 2.3.2. Direct Incorporation of Blackberry Powders (D) into Film-Forming Solution

Blackberry pulp powders (BL) and microencapsulated blackberry pulp powder (ML) were added directly into the film-forming solution and homogenized manually with the aid of a drumstick. Aliquots of 25 mL of resulting solutions were dispensed onto Plexiglas plates (12 cm diameter). Films were dried for about 24 h, at room temperature (25 ± 5 °C). After being removed from the plates, the films were conditioned at 25 °C and 55% ± 3% of relative humidity for 48 h before their characterization.

#### 2.3.3. Incorporation of Blackberry Powder by Sprinkling (S) into Film-Forming Suspension

Aliquots of 25 mL of resulting film-forming solution (solution of arrowroot starch and glycerol obtained according to item 2.3.1) were initially deposited onto Plexiglas plates (12 cm diameter). Blackberry pulp powder (BL) and microencapsulated blackberry pulp powder (ML) were homogeneously sprinkled, through a stainless-steel sieve with 53 mesh, onto all the surface area of the film-forming solution already disposed on the plates [24]. Films were removed from the support plates after drying for 24 h at room temperature (25 ± 5 °C). Films were stored at 25 °C and 55 ± 3% relative humidity for 48 h before their characterization.

### 2.4. Films Characterization

#### 2.4.1. Visual Aspect 

Visual and tactile analyses were performed in order to select the most homogeneous films that were flexible for handling when removed from plates. Films without these characteristics were rejected.

#### 2.4.2. Microstructure 

The morphological characteristics of the surface and the cross section developed from the film samples were examined under a scanning electron microscope (Leo 440i, Electron Microscopy/Oxford, Cambridge, England). Film samples were placed on double-sided carbon adhesive tape adhered to a stub, submitted to the application of a gold layer (model K450, Sputter Coater EMITECH, Kent, UK and observed in a scanning electron microscope operated at 20 kV.

#### 2.4.3. Colour Determination

The colours of the films were measured using a Hunter Lab colorimeter (Color Quest XE 2819, USA). The equipment was set with D65 illuminant and calibrated with a standard white reflector plate. Three films of each treatment were evaluated. The CIELab, mainstream color space coordinate system defined by International Commission on Illumination (CIE) was used for determining a*, b* and L* parameters, where L* is luminosity (L* = 0 black and L* = 100 white), a* is the greenness and redness of samples (+a* = red and -a* = green) and b* represents the blueness and yellowness (+b* = yellow and -b* = blue). Colour difference (ΔE *) between the films were calculated according to Nogueira, Fakhouri and de Oliveira [24].

#### 2.4.4. Anthocyanins Content

The anthocyanins content in the films and powders was determined by the method employed by Sims and Gamon [34], with adaptations. Film samples were previously macerated in liquid nitrogen, weighed in triplicate and homogenized with 3 mL of cold acetone/Tris-HCl solution (80:20, volume/volume, pH 7.8 0.2 M) for 1 min. The samples remained at rest for 1 h, protected from light and centrifuged for 15 min at 3500 rpm. The supernatants were immediately read in a spectrophotometer (B422 model, Micronal) in visible region at 537 nanometers (anthocyanins). The acetone/Tris-HCl solution was used as blank sample. Absorbance values were converted to mg/100 g of blackberry pulp solids.

#### 2.4.5. Antioxidant Capacity

Antioxidant capacity was determined by the ABTS (2.2’-azinobis (3-ethylbenzothiazoline-6-sulfonic acid) method, which estimates the sample’s capacity to isolate ABTS radicals. These ABTS radicals were formed by the reaction of 140 mM potassium persulfate with standard 7 mM ABTS solution, stored in the dark for 16 h at room temperature. Then, ABTS (P.A.) was diluted with ethanol to obtain the absorbance value of 0.70 nm ± 0.05 nm to 734 nm. Samples of the films and powders were extracted in methanol solution (50% methanol in distilled water, *v*/*v*) and then in acetone solution (70% acetone in distilled water, *v*/*v*) to determine their antioxidant capacity. Aliquots of 30 µL of extract were added to 3 mL of ABTS radical and kept in the dark for 6 min. Standard curve was fitted with Trolox [6-hydroxy-2.5.7.8-tetrametilchroman-2-carboxylic acid] at concentrations ranging from 100 to 2000 µM. Results were calculated according to an equation fitted by standard curve and expressed by µg.g^−1^ solid of Trolox equivalent (TE). All of the analyses were performed in triplicate.

#### 2.4.6. Film Thickness, Water Activity and Moisture Content

Film thickness was measured using a micrometre (Mitutoyo brand, model MDC 25 M, MFG/Japan). For each film, thickness was determined by randomly measuring 10 different regions of the film. The moisture content of films was gravimetrically determined, in triplicate, by drying the samples in an air-forced oven 105 ºC for 24 h [29]. The water activity was determined using AquaLab Lite apparatus (Decagon Devices Inc., Pullman, WA, USA), by direct reading of the samples, circularly sized into 35 mm in diameter, in triplicate, at 25 °C. 

#### 2.4.7. Solubility in Water

The method proposed by Gontard, Guilbert, and Cuq [35] was used for determining the water solubility of film samples, which were cut in circles (2 cm of diameter) and weighed (initial dry weight at 105 °C for 24 h). Then, they were placed individually into beakers containing 50 mL of distilled water, and stirred at 75 rpm for 24 h at 25 ± 2 °C. Finally, the samples were removed and dried at 105 °C until constant weight (final dry weight). The solubility of films (%) was calculated as the percentage of total soluble matter. 

#### 2.4.8. Water Vapor Permeability

Water vapor permeability tests were conducted using the ASTM E96-80 method [36]. Film samples were placed, in triplicate, in acrylic permeation cells containing dried calcium chloride (0% relative humidity at 25 °C). These cells were weighed and placed in a desiccator maintained at 25 °C and 75% RH using saturated sodium chloride solution.

Water vapor transferred through the film was determined by the mass gain of calcium chloride. Cells were weighed daily for at least 7 days. For each film sample, the thickness was determined by random measurements of 5 different regions of the film. The water vapor permeation rate (WVP) was obtained following the equation described by Nogueira, Fakhouri and de Oliveira [24]. 

#### 2.4.9. Mechanical Properties

Tensile strength and elongation at break were obtained using a texturometer conducted according to the ASTM standard method D 882-83 [37], with modifications [38]. For each film, six samples were cut in rectangular strips (100 mm × 25 mm). Thickness was determined by randomly measuring 5 different regions of the sample, before analyses. For this test, films were fixed by two distal claws, initially 50 mm apart, which moved at the speed of 1 mm/s. Tensile strength (MPa) was calculated by dividing the maximum force at the moment of rupture (N) by cross-sectional area of the film (m^2^). Elongation at break (%) was calculated dividing the difference between the distance at the moment of rupture (cm) and the initial separation distance (cm) by the distance at the moment of rupture (cm), multiplied by 100. 

### 2.5. Statistical Analysis

Significant differences between average results were evaluated by analysis of variance (ANOVA) and Tukey test at 5% level of significance, using SAS software (Cary, NC, USA).

## 3. Results and Discussion

### 3.1. Characterization of Blackberry Powder

**Figure 1:**
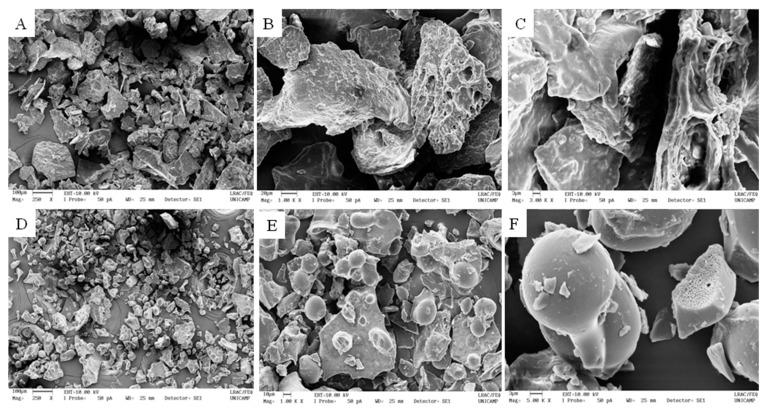
Scanning electron micrographs of freeze-dried blackberry pulp (BL, images **A**, **B** and **C**) and freeze-dried microencapsulated blackberry pulp (ML, images **D**, **E** and **F**) powders: images (**A**,**D**) 250× magnification, (**B**,**E**) 1000× magnification, (**C**,**F**) 3000 × magnification.

Figure 1 shows microstructure of BL and ML powders observed through SEM images. External morphology of freeze-dried BL and ML resembled a broken glass structure of variable sizes, with typical folds, slight cracks and porosity on the surface due to the loss of water content during the freeze-drying process. Similar characteristics were observed by Yamashita [21] and Franceschinis, Salvatori, Sosa and Schebor [39] for freeze-dried blackberry (*Rubus* spp.) powder.

Furthermore, for ML powder, it was possible to observe spherical particles distributed throughout their vitreous structures. It is believed that these particles are the result of the microencapsulation of blackberry pulp by the mixture of arrowroot starch and arabic gum used as an encapsulating agent. Shi et al. [40] produced nanoparticles of starch by spray-drying and freeze-drying methods. Nanoparticles of starch produced by both methods were spherical and showed very similar morphology.

Table 1 shows the results of the characterization of blackberry pulp (BL) and microencapsulated blackberry pulp (ML) powders obtained by freeze-drying. The yield obtained from the blackberry pulp’s drying process, with or without encapsulating agents, was high. BL and ML powders presented low water content and the water activity was less than 0.3, indicating that they are biochemically and microbiologically stable because below that value (Aw < 0.3) there are interruptions in non-enzymatic reactions and there is no growth of microorganisms [41].

ML powder was significantly (*p* < 0.05) less hygroscopic and soluble than BL powder. This fact happened because arrowroot starch and arabic gum are materials with low hygroscopicity; consequently, the microencapsulation of blackberry pulp tends to reduce the hygroscopicity of resulting powders. Besides, although arabic gum is highly soluble [42], arrowroot starch in its native form presents low solubility in water at room temperature. This fact probably contributed to the decrease in the solubility of the blackberry powder.

Although microencapsulation is a widely used method to protect bioactive compounds against adverse environmental conditions, such as pH, light and oxygen [18], no significant differences (*p* > 0.05) were observed between the values of anthocyanins and antioxidant properties for BL and ML powders. Since freeze-drying is a method that does not use high temperatures during the drying process and is based on dehydration by the sublimation of a frozen product, these factors probably contributed to the maintenance of bioactive compounds and antioxidant capacity [21].

The resulting powders presented a reddish colour, typical of blackberry pulp. ML powder showed significantly (*p* < 0.05) lighter coloration than BL powder, due to the presence of an encapsulating agent which has a lighter colour.

### 3.2. Films Characterization

#### 3.2.1. Visual Aspect 

**Figure 2:**
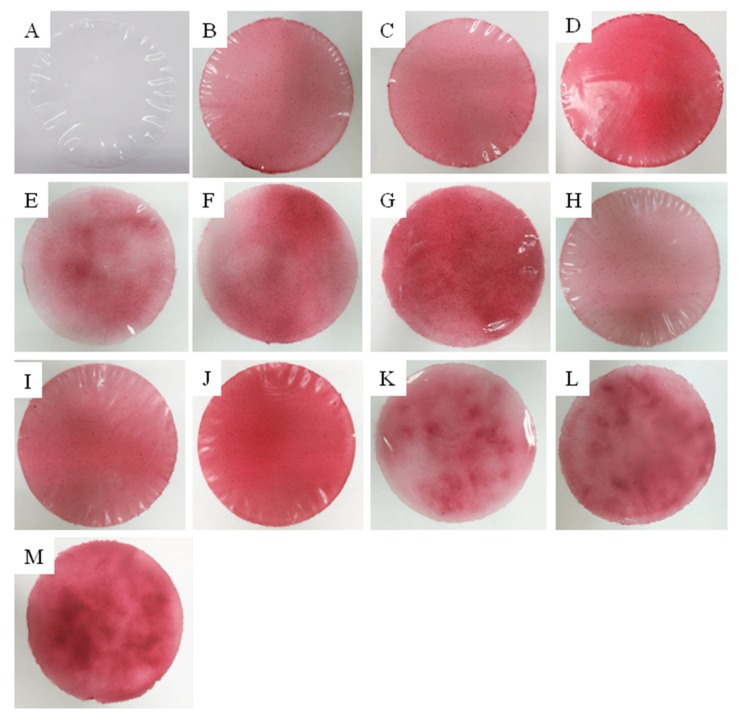
Photographic images of the films samples with 0%, 20%, 30% and 40% of freeze-dried blackberry pulp (BL) and freeze-dried microencapsulated blackberry pulp (ML) incorporated directly (D) and by sprinkling (S): (**A**) 0%; (**B**) 20% BLD; (**C**) 30% BLD; (**D**) 40% BLD; (**E**) 20% BLS; (**F**) 30% BLS; (**G**) 40% BLS; (**H**) 20% MLD; (**I**) 30% MLD; (**J**) 40% MLD; (**K**) 20% MLS; (**L**) 30% MLS; (**M**) 40% MLS.

The incorporation of blackberry powders into the film-forming solution gave the arrowroot starch films a reddish colour, which can be observed in Figure 2. In larger concentrations, the colour was more visually remarkable. All films could be removed from the plates and, in general, had good appearance and transparency. Films with 0%, 20%, 30% and 40% of blackberry pulp powder (BLD) and microencapsulated blackberry pulp powder (MLD) directly incorporated were visually homogeneous, continuous and very flexible for handling. On the other hand, films with blackberry powders (BL and ML) incorporated by sprinkling (S) were brittle and sensitive to handling. 

#### 3.2.2. Microstructure

**Figure 3:**
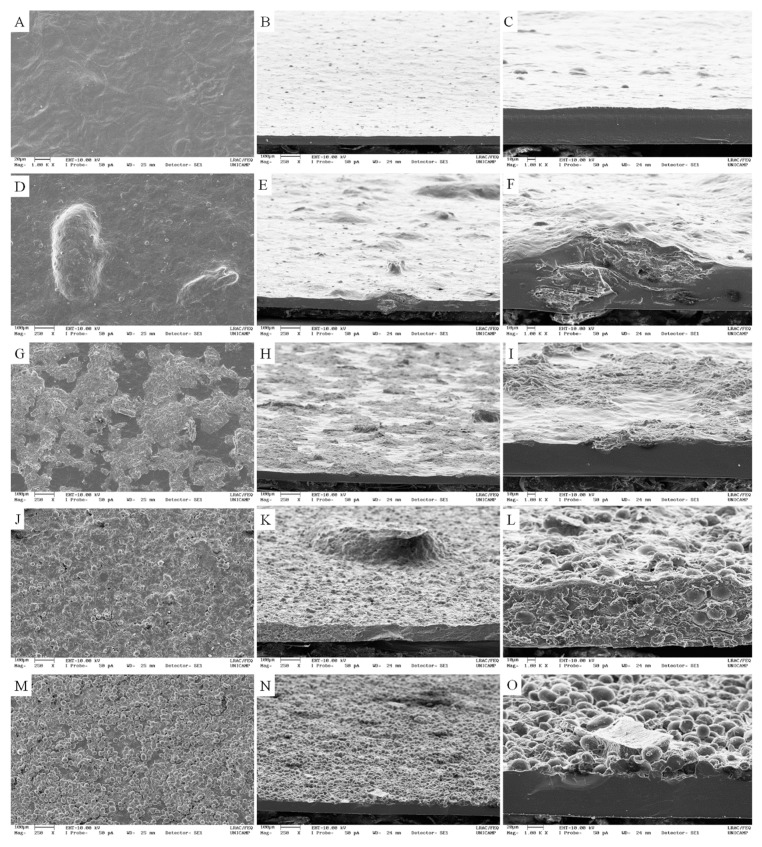
Scanning electron microscope (SEM) images of the films with 0% and 40% of freeze-dried blackberry pulp (BL) and freeze-dried microencapsulated blackberry pulp (ML) incorporated directly (D) and by sprinkling (S): (**A**) surface of control film; (**B**,**C**) cross section of control film; (**D**) surface of 40% BLD film; (**E**,**F**) cross section of 40% BLD film; (**G**) surface of 40% BLS film; (**H**,**I**) cross section of 40% BLS film; (**J**) surface of 40% MLD film; (**K**,**L**) cross section of 40% MLD film; (**J**) surface of 40% MLS film; (**K**,**L**) cross section of 40% MLS film. Images (**A**), (**C**), (**F**), (**I**), (**L**) and (**O**) with 250× magnification and images (**B**), (**D**), (**E**), (**G**), (**H**), (**J**), (**K**), (**M**) and (**N**) with 1000× magnification.

The morphological characteristics of the films can be observed in Figure 3. Differently from the film with 0%, which presented organized polymer matrix and regular surface (Figure 3A–C), films developed with 40% of blackberry powders presented rough surfaces and morphological characteristics directly associated to those observed in BL and ML powders. Shi et al. (2013) also observed that the incorporation of starch nanoparticles obtained by spray drying and freeze-drying made the surface of starch film rough. According to the authors, protuberances found on the film surface resulted from the presence of starch nanoparticles. The structural characteristics exhibited by the resulting films also varied according to the type of technique, directly or by sprinkling, employed in the incorporation of the blackberry powders into the film-forming solution. The cross-section images of films incorporated directly with 40% BL and ML (Figure 3D–F,J–L, respectively) revealed the presence of dispersed and agglomerated blackberry particles within the starch network, which resulted in organised and disorganised regions.

This result may have been obtained due to the poor dispersion of powders in high concentrations in the film-forming solution, given their high viscosity. Similar characteristics were observed by Sartori and Menegalli [43] in film containing solid lipid microparticles. Castillo et al. [44] observed randomly dispersed nano-agglomerates and individual platelets of talc in nanocomposite films by transmission electron microscopy (TEM). Mukurumbira, Mellem and Amonsou [25] observed that the incorporation of nanocrystals into starch films made their surfaces irregular and rough. According to the authors, these changes could be attributed to the presence of aggregated nanocrystals and, possibly, the interactions between nanocrystals and amylose in the starch. Ortega, Giannuzzi, Arce and García [45] incorporated silver nanoparticles into starch films and also observed the presence of agglomerates of nanoparticles in the gelatinized starch suspension. 

For films embedded with sprinkled blackberry powder it was observed that the use of a sieve to sprinkle BL and ML powders over the film-forming solution allowed them to become adhered to the film surface after drying. In addition, as can be seen in Figure 3G–I, the ML particles penetrated more into the starch matrix than the BL particles, which tended to remain mostly on the surface of the matrix. Probably, ML particles are denser than BL (Figure 3M–O).

#### 3.2.3. Colour Determination

Table 2 presents colour parameters of film samples with 0% [24], 20%, 30% and 40% of blackberry pulp (BL) and microencapsulated blackberry pulp (ML) incorporated directly (D) and by sprinkling (S). Films incorporated with blackberry exhibited colorimetric parameters correlated with those found for BL and ML powders (Table 1), differing significantly from the film with 0% [24] which was colourless. 

The results indicate that the incorporation and concentration of BL and ML significantly (*p* < 0.05) affected chromaticity parameters (a* and b*) of films. With the incorporation of BL and ML into the film with 0% [24], values of b* increased, varying from negative to positive, showing a propensity for yellow coloration. Similarly, Li et al. [46] observed that the addition of starch nanocrystals resulted in the yellowing of pea starch films. 

A relevant increase in values of a* was also observed, evidencing a tendency for red coloration and the presence of anthocyanins pigments. It was reported that a* coordinate is attributed to the anthocyanins content in blackberry, which is responsible for the red colour of BL and ML powders [21,47]. Once again, low values indicate that films developed with BL and ML powders exhibited colour shades ranging from red to yellow-orange, correlating with film visual observations.

Ortega et al. [45] also observed that the incorporation and concentration of silver nanoparticles significantly affected chromaticity parameters (a* and b*); although, in both cases the corresponding values were very low and, visually, nanocomposite films remained colourless. 

As expected, the incorporation of BL and ML powders and their concentration also significantly affected (*p* < 0.05) colour difference (DE). The increase from 20% to 40% in the concentration of BL and ML incorporated directly and by sprinkling into arrowroot starch film (0% [24]) caused a statistically significant increase (*p* < 0.05) in a* and b* values, leading to a decrease in luminosity L* and increase in total colour difference ΔE* (Table 2). 

#### 3.2.4. Anthocyanins Content and Antioxidant Capacity

Table 3 shows anthocyanins content (mg/100 g of blackberry solids) and antioxidant capacity (μmol of Trolox/g of blackberry solids) of films after the drying process. In the absence of blackberry powder (BL and ML), arrowroot starch film showed an insignificant amount of anthocyanins content and antioxidant capacity. Thus, it is clear that the anthocyanins content and antioxidant capacity presented by arrowroot starch films incorporated with BL and ML are directly related to their content in blackberry powders (Table 1).

It is important to add that there was a decrease in the anthocyanins content of films with BL and ML powders when compared with the initial amount of the respective powders. This happened because anthocyanins present great susceptibility to degradation when exposed to environmental factors such as temperature, light, pH and oxygen [48] during film production, resulting in their decrease in dried films. Maniglia, Tessaro, Lucas and Tapia-Blácido [49] also reported losses of phenolic compounds due to a possible oxidative degradation of phenolic groups caused by heating applied during the preparation of film-forming solution and during the film-drying process. Nevertheless, in this research work, increasing the concentration of blackberry incorporated into the film led to a slight increase in anthocyanins content.

In scientific literature there are several works reporting a high correlation between phenolic compound content and antioxidant capacity [50,51]. However, increasing the concentration from 20% to 40% BL and ML powders incorporated into the film-forming solution has shown a tendency to increase the anthocyanins content in the resulting films. This same trend was not observed for the antioxidant capacity. A similar behaviour was observed by Nogueira et al. [52] for arrowroot starch films incorporated with blackberry pulp, and by Chang-Bravo, López-Córdoba, and Martino [53] for extracts of yerba mate and propolis. According to Chang-Bravo, López-Córdoba, and Martino [53], for both extracts, DPPH(2,2-Diphenyl-1-picryl-hidrazil) radical scavenging activity increased proportionally to their polyphenols content until reaching a plateau in which the antioxidant capacity became independent from the concentration.

As for the antioxidant capacity, the type of incorporation of BL and ML powders, either directly or by sprinkling, had more influence than the variation in their concentration in films. The films incorporated with 30% and 40% of BL and ML by sprinkling had the highest antioxidant capacity. The fact that the particles of BL and ML remained on the surface of the film probably facilitated the extraction of bioactive compounds, due to a greater number of particles in direct contact with the extraction solvent, which consequently generated greater antioxidant activity. However, in direct incorporation, the BL and ML powders integrated into the polymer matrix, which probably reduced their surface area when in direct contact with extraction solvents. This fact may have hindered the extraction of bioactive compounds and, consequently, generated lower antioxidant capacity.

Moreover, it is important to emphasize that there are great varieties of bioactive compounds with antioxidant capacity in fruit extracts [50]. In addition to anthocyanins, other bioactive phenolic acids, tannins and ascorbic acid [14] may be also present in films with blackberry, contributing to the total antioxidant capacity of BL and ML powders.

#### 3.2.5. Water Activity and Moisture Content

Water activity and moisture content values of films are shown in Table 4. The water activity obtained for the control film (0% [24]) and films containing BL and ML ranged from 0.37 to 0.55. Films can be considered stable against microbial proliferation. According to Quek, Chock and Swedlund [54], in general, food with aw < 0.6 is considered microbiologically stable and, in case of any spoilage, it is induced by chemical reactions rather than by microorganisms.

Moisture content in the control film (0% [24]) and in films containing BL and ML ranged from 7.88% to 13.65%. Similar results were found for films made of amadumbe and potato starch with amadumbe starch nanocrystals (0, 2.5, 5 and 10%), which presented moisture content ranging from 9.3% to 13.4% and 14.8% to 16.7%, respectively [25].

The incorporation of BL and ML powders by sprinkling (S) led to a significant decrease (*p* < 0.05) in the moisture content of films in comparison with those incorporated directly and the control films (0% [24]). Li et al. [46] observed a decrease in moisture content with the incorporation of starch nanocrystals into pea starch films. In this study, the impact of particles falling onto the film-forming solution by sprinkling may have triggered a discontinuity of polymer matrix on its surface, favouring water release from the starch structure. Low water content in BLS and MLS films may be one of the possible causes of brittleness and fragility of these films.

#### 3.2.6. Film Thickness, Water Solubility, Water Vapor Permeability and Mechanical Properties

Table 5 shows thickness (mm), water solubility (%), water vapor permeability (g.mm/m^2^.day.kPa), tensile strength (MPa) and elongation at break (%) of films with 0% (0%, [24]), 20%, 30% and 40% of BL and ML powders incorporated directly (D) and by sprinkling (S).

Water solubility, water vapor permeability and mechanical properties are directly influenced by film thickness, among other factors [45]. Thickness of films (0%, [24]) made from arrowroot starch and of films incorporated with BL and ML powders ranged from 0.065 mm (0% [24]) to 0.173 mm (40% MLS). The increase of 20% to 40% in the concentration of BL and ML, incorporated directly and by sprinkling into the arrowroot starch matrix (0% [24]), caused a statistically significant increase (*p* < 0.05) in the thickness of the control film (0%, [24]). This increase in thickness is due to the increase in solid content, since the same volume of the film-forming solution was used in the plate area. Besides, the agglomeration of blackberry particles in the polymer matrix, evidenced by prominences on the surface of films by SEM (Figure 3), may have contributed to the increase in film thickness. Ortega et al. [45] also observed that silver nanoparticles caused a slight increase in film thickness. The authors also attributed these results to the increased solid content and possible agglomeration of silver nanoparticles. 

Water solubility, water vapor permeability and mechanical property are important parameters for choosing the application of biopolymer films [25,55,56,57,58,59]. Some applications, considering the potential use of these new polymer films instead of synthetic packaging, may require low water solubility, strength and flexibility to tolerate the typical effort made by packaging materials during handling and food transportation, maintaining the integrity of the product [25,55,56]. Some other applications, such as encapsulation, may require significantly higher solubility [25,55] in order to allow release of the encapsulated material into the surroundings.

The type of incorporation and the variation of BL and ML concentrations had significant effect (*p* < 0.05) on the solubility, tensile strength and elongation at break of starch films. In general, water solubility increased significantly (*p* < 0.05) while tensile strength decreased after increasing the concentrations of BL and ML powders (20%, 30% and 40%) in films, in comparison with the control film (0%, [24]), by direct incorporation. This behaviour could be attributed to a possible reduction of intermolecular attraction forces caused by agglomerations of BL and ML [25,44,45], which led to the disrupting and discontinuity of starch matrix [23] (Figure 3). Consequently, the polymer network was less dense, facilitating water permeation in its structure and solubilization, and reducing its resistance and increasing flexibility. Films prepared with babassu mesocarp flour and starch isolated from babassu mesocarp by casting exhibited similar behaviour [49].

Films incorporated directly with BL and ML were less water soluble than films incorporated with blackberry powder by sprinkling. The location of blackberry particles probably influenced this behaviour. As showed in Figure 3, blackberry powder particles incorporated by sprinkling tended to remain on the surface of the film, unlike directly incorporated particles, which were introduced into the polymer starch network. The fact that the particles of blackberry powder stayed on the surface of the films is believed to have enabled a greater and direct contact of these particles with the water. Additionaly, the fact that the particles are porous and hydrophilic probably led to their solubilization and the formation of holes on the surface of the films, which allowed water molecules to go inside the starch matrix and then its solubilization [23]. The same phenomenon of increased water absorption was observed in the chitosan/starch films with halloysite nanotubes, which was attributed to the increased porosity and hydrophilicity of the nanocomposite films [60]. The addition of hydrophilic clay into kafirin films also affected the hydrophilicity of the film due to the presence of Si-OH groups [61].

As equally observed for water solubility, water vapor permeability presented by films was also significantly affected (*p* < 0.05) by the incorporation of blackberry powder, as well as by the variation in its concentration. According to Ludueña, Vázquez, and Alvarez [62], the passage of water molecules through a polymeric material is the balance between three principal mechanisms: film crystallinity, tortuous pathways through the polymeric matrix, and the presence of structural defects on the surface.

Films with only 20% of powder presented decreasing water vapor permeability in relation to the film with 0% (0%, [24]), as previously reported by Shi et al. [40] for starch films containing spray dried and vacuum freeze-dried starch nanoparticles. At low concentrations, BL and ML powders were easily dispersed into the film-forming solution, increasing compactness of the films, which may have hindered the passage of water molecules [40,46,63]. It is also possible that the presence of blackberry powder particles within the starch matrix, as well as on the surface, introduced a tortuous path for the passage of water molecules, which may have led to a decreasing behaviour in water vapor permeability. A similar result regarding a decrease in water vapor permeability was observed when chitin nano-whiskers (CNWs) was incorporated into the maize starch–based films [64]. 

In a single polymer film, the diffusible molecules migrate from a straight (middle) path that is perpendicular to the film surface. Whereas, in films with nanocomposites, the diffusion molecules must navigate through tortuous paths due to the presence of particles or platelets, as well as through interfacial zones of different permeability characteristics in comparison with those with pure polymer [64,65,66,67,68,69]. In theory, the longer the diffusive pathway of the penetrant, water molecules in this case, the lower the permeability [25,63]. 

However, when more than 20% of BL and ML powders were incorporated directly, the particles tended to form aggregations, as observed in the microstructure of 40% BLD and MLD films (Figure 3). The aggregation of BL and ML particles reduces the interaction between the active surface area and the polymer matrix. This fact tends to weaken their adhesion to the starch matrix interface, destroying the orderly structure of the film to 0% , increasing water vapor permeability [25,64], and following the same tendency observed for water solubility.

## 4. Conclusions

This study has developed a new understanding of the influence of the incorporation of blackberry particles obtained by freeze-drying into the properties of arrowroot starch-based films. The addition of blackberry pulp particles causes interactions with the film matrix, inducing changes in the properties of films. These interactions alter the microstructure, as well as the chemical, mechanical, and barrier properties of the films. It was found that the incorporation of blackberry pulp particles does not only make the surface of arrowroot starch rough and irregular, but also thicker, more flexible, soluble in water, and less mechanically resistant. Additionally, blackberry pulp particles transferred colour, anthocyanins and antioxidant capacity to the arrowroot starch film.

Properties presented by the resulting films were also influenced by the concentration and the type of method used, direct or by sprinkling, for the incorporation of blackberry powders into the film-forming solution. Films with only 20% BL and ML presented lower water vapor permeability rates than the film with 0%. This behaviour was attributed to a better dispersion of blackberry powder at low concentrations in the film-forming solution, as well as to the introduction of tortuous paths in the starch matrix. At concentrations above 30%, there was an increase in water vapor permeability due to the presence of agglomerated blackberry powder particles. Films incorporated with blackberry powder by sprinkling had higher antioxidant capacity and were more soluble in water, showing great potential to be used as a vehicle for releasing bioactive compounds into the surroundings.

## Figures and Tables

**Table 1:** Characterization of blackberry powder obtained by freeze-drying.

Analysis	Blackberry Pulp (BL)	Microencapsulated Blackberry Pulp (ML)
Process yield (%)	89.24 ± 2.81 ^a^	95.86 ± 0.89 ^a^
Moisture content (%)	10.72 ± 2.81 ^a^	4.50 ± 0.31 ^a^
Aw (decimal)	0.13 ± 0.01 ^a^	0.11 ± 0.01 ^b^
Hygroscopicity (g of adsorbed water/100 g solids)	21.28 ± 0.45 ^a^	12.86 ± 0.1 ^b^
Solubility in water (%)	61.26 ± 0.49 ^a^	53.84 ± 0.76 ^b^
Total Anthocyanins (mg/100 g of blackberry solids)	125.27 ± 9.77 ^a^	125.99 ± 5.25 ^a^
ABTS radical (µmol of Trolox/g of blackberry solids)	288.43 ± 30.70 ^a^	309.18 ± 34.09 ^a^
Color		
L*	47.29 ± 2.35 ^b^	57.23 ± 1.57 ^a^
a*	14.18 ± 2.97 ^a^	20.13 ± 1.17 ^a^
b*	4.95 ± 0.56 ^a^	3.59 ± 0.08 ^a^

Same letters in the same line show no statistical difference (*p* > 0.05).

**Table 2:** Colour parameters of film samples with 0%, 20%, 30% and 40% of freeze-dried blackberry pulp (BL) and freeze-dried microencapsulated blackberry pulp (ML) incorporated directly (D) and by sprinkling (S). Results of the control film characterization (0%) were published by Nogueira, Fakhouri and Oliveira [24].

Films	L*	a*	b*	ΔE*
0%	91.54 ± 1.16 ^a^	1.96 ± 0.09 ^g^	-8.64 ± 0.46 ^i^	-
20% BLD	63.16 ± 0.40 ^b^	23.50 ± 0.18 ^e^	1.35 ± 0.09 ^hg^	37.00 ± 0.43 ^de^
30% BLD	62.16 ± 0.48 ^b^	24.60 ± 0.30 ^dce^	1.69 ± 0.06 ^g^	38.50 ± 0.56 ^de^
40% BLD	47.28 ± 0.66 ^edf^	35.05 ± 0.36 ^a^	4.30 ± 0.16 ^ba^	56.76 ± 0.76 ^a^
20% BLS	57.89 ± 3.68 ^cb^	27.44 ± 2.88 ^c^	0.44 ± 0.29 ^h^	43.17 ± 4.62 ^dc^
30% BLS	46.67 ± 1.51 ^edf^	34.31 ± 0.85 a	1.86 ± 0.36 ^fg^	56.30 ± 1.75 ^ba^
40% BLS	40.72 ± 3.87 ^gf^	35.92 ± 0.61 ^a^	3.52 ± 1.15 ^bdc^	62.33 ± 3.70 ^a^
20% MLD	62.78 ± 1.54 ^b^	19.28 ± 0.68 ^f^	3.11 ± 0.31 ^dec^	35.57 ± 1.68 ^e^
30% MLD	53.84 ± 0.37 ^cd^	24.27 ± 0.24 ^de^	4.09 ± 0.07 ^bac^	45.61 ± 0.43 ^c^
40% MLD	42.70 ± 0.70 ^gf^	30.44 ± 0.11 ^b^	4.72 ± 0.08 ^a^	58.09 ± 0.66 ^a^
20% MLS	49.89 ± 1.03 ^ed^	25.98 ± 0.34 ^dce^	2.21 ± 0.39 ^feg^	49.29 ± 0.95 ^bc^
30% MLS	42.96 ± 2.19 ^egf^	30.62 ± 0.28 ^b^	2.90 ± 0.35 ^fde^	57.58 ± 1.93 ^a^
40% MLS	36.27 ± 0.13 ^g^	26.82 ± 1.50 ^dc^	3.74 ± 0.29 ^bdac^	61.94 ± 4.85 ^a^

Same letters in the same column show no statistical difference (*p* > 0.05).

**Table 3:** Anthocyanins content and antioxidant capacity of films with 0%, 20%, 30% and 40% of freeze-dried blackberry pulp (BL) and freeze-dried microencapsulated blackberry pulp (ML) incorporated directly (D) and by sprinkling (S). Results of the control film characterization (0%) were published by Nogueira, Fakhouri and Oliveira [24].

Films	Total Anthocyanins (mg/100 g of Blackberry Solids)	ABTS (μmol of Trolox/g of Blackberry Solids)
0% *	0.32 ± 0.12 ^e^	9.15 ± 6.51 ^f^
20% BLD	47.53 ± 6.06 ^cd^	161.99 ± 10.54 ^e^
30% BLD	40.23 ± 1.29 ^cd^	180.68 ± 22.48 ^ed^
40% BLD	76.47 ± 0.98 ^a^	174.24 ± 51.73 ^ed^
20% BLS	70.01 ± 9.65 ^ba^	253.57 ± 24.68 ^ed^
30% BLS	71.63 ± 6.96 ^ba^	368.32 ± 37.02 ^bac^
40% BLS	81.95 ± 12.83 ^a^	408.24 ± 32.04 ^a^
20% MLD	38.13 ± 0.55 ^d^	272.64 ± 73.00 ^dc^
30% MLD	41.79 ± 0.10 ^cd^	274.55 ± 46.66 ^dc^
40% MLD	39.39 ± 9.41 ^cd^	278.93 ± 8.32 ^bdc^
20% MLS	45.47 ± 2.13 ^cd^	385.62 ± 18.54 ^ba^
30% MLS	56.09 ± 1.22 ^bc^	436.78 ± 24.48 ^a^
40% MLS	55.68 ± 1.63 ^bcd^	446.82 ± 39.66 ^a^

Same letters in the same column show no statistical difference (*p* > 0.05). * For 0% [24] film, anthocyanins content and antioxidant capacity is expressed by total solids, Total Anthocyanins (mg/100 g of total solids) and ABTS (μmol of Trolox/g of total solids).

**Table 4:** Water activity and moisture content of films with 0%, 20%, 30% and 40% of freeze-dried blackberry pulp (BL) and freeze-dried microencapsulated blackberry pulp (ML) incorporated directly (D) and by sprinkling (S). Results of the control film characterization (0%) were published by Nogueira, Fakhouri and Oliveira [24].

Films	Aw at 25 °C	Moisture Content (%)
0%	0.43 ± 0.05 ^bc^	11.30 ± 0.10 ^bdc^
20% BLD	0.37 ± 0.01 ^c^	9.94 ± 1.02 ^fedg^
30% BLD	0.37 ± 0.01 ^c^	10.89 ± 0.62 ^bedc^
40% BLD	0.40 ± 0.01 ^bc^	13.65 ± 1.00 ^a^
20% BLS	0.55 ± 0.09 ^a^	8.72 ± 0.79 ^fhg^
30% BLS	0.45 ± 0.03 ^bc^	8.50 ± 0.84 ^hg^
40% BLS	0.41 ± 0.02 ^bc^	9.97 ± 1.17 ^fedg^
20% MLD	0.40 ± 0.01 ^bc^	10.42 ± 0.13 ^fedc^
30% MLD	0.38 ± 0.01 ^c^	12.14 ± 0.38 ^bac^
40% MLD	0.39 ± 0.01 ^c^	12.30 ± 0.83 ^ba^
20% MLS	0.42 ± 0.01 ^bc^	8.18 ± 0.28 ^hg^
30% MLS	0.47 ± 0.03 ^ba^	7.88 ± 0.72 ^h^
40% MLS	0.45 ± 0.02 ^bc^	9.22 ± 0.50 ^fehg^

Same letters in the same column show no statistical difference (*p* > 0.05).

**Table 5:** Thickness (mm), water solubility (%), water vapour permeability (g.mm/m^2^.day.kPa), tensile strength (MPa) and elongation at break (%) of films with 0%, 20%, 30% and 40% of freeze-dried blackberry pulp (BL) and the freeze-dried microencapsulated blackberry pulp (ML) incorporated directly (D) and by sprinkling (S). Results of the control film characterization (0%) were published by Nogueira, Fakhouri and Oliveira [24].

Films	Thickness (mm)	Solubility in Water (%)	Permeability to Water Vapor (gmm/m^2^daykPa)	Tensile Strength (MPa)	Elongation at Break (%)
0%	0.065 ± 0.005 ^d^	14.18 ± 0.26 ^g^	3.62 ± 0.27 ^hdfge^	22.71 ± 1.27 ^a^	3.18 ± 0.44 ^d^
20% BLD	0.092 ± 0.005 ^dc^	21.64 ± 0.93 ^fe^	3.03 ± 0.10 ^hfge^	3.60 ± 0.33 ^ih^	23.53 ± 3.60 ^a^
30% BLD	0.121 ± 0.014 ^bdaac^	22.76 ± 1.13 ^dfe^	6.63 ± 0.39 ^bc^	3.55 ± 0.12 ^ih^	23.33 ± 0.72 ^a^
40% BLD	0.154 ± 0.054 ^bac^	26.14 ± 1.16 ^dce^	5.40 ± 0.47 ^dce^	2.73 ± 0.33 ^i^	26.42 ± 1.40 ^a^
20% BLS	0.082 ± 0.006 ^d^	19.26 ± 1.68 ^fe^	1.67 ± 0.12 ^h^	10.84 ± 1.69 ^b^	7.46 ± 2.55 ^c^
30% BLS	0.098 ± 0.013 ^bdc^	24.65 ± 1.95 ^dce^	2.38 ± 0.45 ^hg^	8.16 ± 0.64 ^dc^	5.26 ± 1.55 ^dc^
40% BLS	0.113 ± 0.016 ^bdac^	27.98 ± 2.69 ^bc^	3.47 ± 0.14 ^hfge^	6.32 ± 0.85 ^fe^	18.32 ± 4.69 ^b^
20% MLD	0.150 ± 0.024 ^bac^	21.74 ± 1.70 ^fe^	2.43 ± 0.36 ^hg^	7.02 ± 0.99 ^de^	3.99 ± 0.76 ^dc^
30% MLD	0.146 ± 0.022 ^bac^	22.18 ± 0.36 ^dfe^	7.80 ± 0.07 ^ba^	5.62 ± 0.40 ^feg^	3.28 ± 0.42 ^d^
40% MLD	0.154 ± 0.010 ^ba^	23.69 ± 0.77 ^dfce^	9.23 ± 0.47 ^a^	4.51 ± 0.29 ^hg^	7.72 ± 0.50 ^c^
20% MLS	0.147 ± 0.017 ^bac^	27.14 ± 2.45 ^dc^	4.42 ± 0.17 ^dfge^	8.87 ± 0.86 ^c^	4.32 ± 0.40 ^dc^
30% MLS	0.153 ± 0.005 ^bac^	33.89 ± 2.50 ^a^	5.08 ±1.87 ^dfce^	6.98 ± 0.60 ^de^	3.96 ± 0.71 ^dc^
40% MLS	0.173 ± 0.011 ^a^	32.33 ± 1.39 ^ba^	5.57 ± 1.05 ^dc^	4.82 ± 0.67 ^fhg^	3.25 ± 0.96 ^d^

Same letters in the same column show no statistical difference (*p* > 0.05).
